# Supplementary material for: Modelling cytoskeletal transport by clusters of non-processive molecular motors with limited binding sites
Source: R Soc Open Sci. 2020 Aug 5;7(8):200527. doi: 10.1098/rsos.200527 (PMC7481682; doi:10.1098/rsos.200527)
Supplement: Appendices [file rsos200527supp1.pdf]

Modelling cytoskeletal transport by clusters of non-processive molecular motors with limited binding sites

Naruemon Rueangkham, Ian D. Estabrook, Rhoda J. Hawkins

Appendices

A. PROCESSIVE MOTORS GILLESPIE ALGORITHM

The Gillespie algorithm is the method where the time until the next event $\mathrm{d}t$ is drawn from an exponential distribution with rate parameter given by the sum of the rates of all events possible from the current state. The main concept is that one, and only one, event happens each time step and that the duration of each time step changes. The leading motor can move forward and backward with rates p_1 and q_1 and the following motors with the rates p and q, respectively. Therefore, there are four possible events, however, due to the simple exclusion process, not all these events will be available in each time step. The time until the next event is calculated each time step as $\tau = \frac{1}{\alpha} \ln \frac{1}{r_1}$ where α is the total probability of all events possible in that time step and r_1 is a random number from a uniform distribution $(0, 1)$. Which event happens in that time step is determined by a second random number, r_2, drawn from the uniform distribution.

The leading motor's velocity for different number, N, of bound motors from our Gillespie simulations is shown in figure S16 (a) and (b). Our simulation results using the Gillespie algorithm match those using our fixed time step method within the error bars. The error bars for the Gillespie algorithm are so small as to be barely visible in figure S16 since they lie inside those of our fixed time step method. Given the equivalence of our results using each method we are satisfied to use the faster fixed time step method to present our results in the main text.

B. NON-PROCESSIVE MOTORS GILLESPIE ALGORITHM

We also adapt the Gillespie algorithm from processive motors to non-processive model drawn in the main text figure 5. Including the binding on and off the filament means we now have six possible events to include in the total probability α with the addition of nk_{off} and $(N-n)k_{\mathrm{on}}$

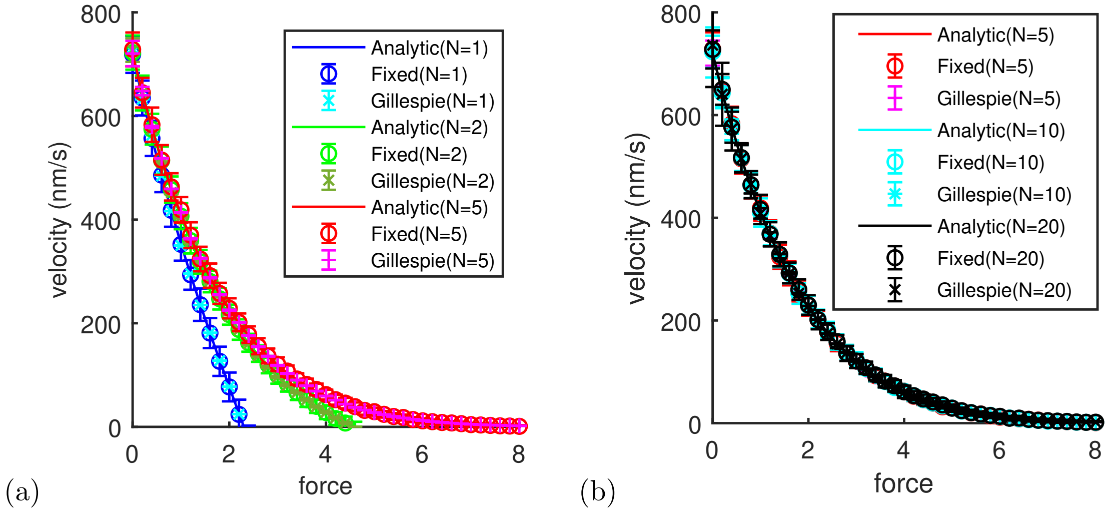

Figure S16: Force-velocity curves of processive motors from simulations using the Gillespie algorithm (star symbols) compared to our fixed time step Monte Carlo algorithm (circles) for (a) a small motor number ($N = 1, 2$ and 5) and (b) for a large motor number ($N = 5, 10$ and 20) compared with the analytical solution (lines) given by equation (2.2) with parameters $p = 100\,\mathrm{s}^{-1}$, $q = 10\,\mathrm{s}^{-1}$, $\delta = 0.5$. The force on the x-axis is the dimensionless force $f = F\mathrm{d}x/k_B T$ where F is the physical force and $\mathrm{d}x = 8\,\mathrm{nm}$ is the step size.

for the probabilities of binding off or on respectively. The rest of the algorithm is the same as that described in section A. We compare this algorithm with our results from our fixed time step Monte Carlo simulations described in the main text section 3.3.1 for scenario B (variable number of binding sites). We performed all the modelling details in the same way as for our fixed time step simulations. In scenario B, the number of accessible binding sites changes with time according to the positions of motors already bound in front or behind, as discussed in the main text section 3.3.1. This preserves the sequence of the motors, preventing them from overtaking each other and reflects the idea that their sequence is determined by their attachments to the cargo. This sequence preservation feature is not captured in our mathematical expressions in the main text section 3.1.

We compare the velocity for scenario B (variable number of binding sites) simulated using our fixed time step Monte Carlo and Gillespie algorithm at different forces in figure S17. The results for the velocity from our fixed time step Monte Carlo in figure S17 are consistent with the Gillespie algorithm within the error bars. As expected the smaller error bars in figure S17 show

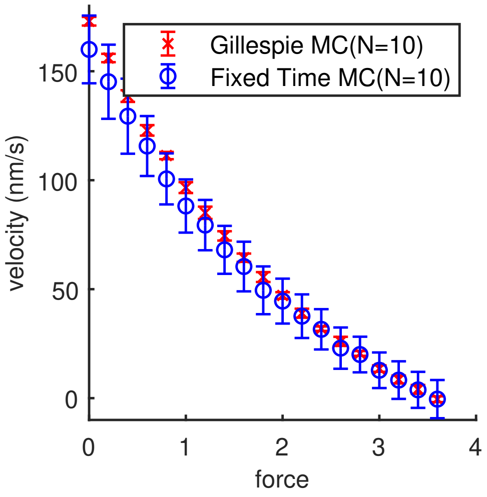

Figure S17: Velocity of a cluster of $N = 10$ non-processive motors from our fixed time step (o) and Gillespie simulations (x) with error bars. The parameters we used are $k_{\text{on}} = 20\ \text{s}^{-1}$, $k_{\text{off}} = 10\ \text{s}^{-1}$, $p = 22\ \text{s}^{-1}$, $q = 2.2\ \text{s}^{-1}$ and $\delta = 0.5$.

that the Gillespie algorithm is more accurate. However, in our case the Gillespie algorithm takes ten times longer to run than our fixed time step Monte Carlo simulations. Therefore in the main text we present results using our fixed time step Monte Carlo simulations since they are more time efficient and sufficiently accurate for our purposes.

C. SEQUENCE PRESERVATION

In figure S18 we present probability distributions for the case of variable number of binding sites (scenario B) but without sequence preservation for both extremes of force $f = 0$ and $f = f_s$. In the simulations presented in this section the simple exclusion process ensures sequence preservation for bound motors stepping but motors can swap positions by unbinding and rebinding. This is in contrast to the scenario B case in the main text section 3.3 in which motors are constrained to rebind in a way that preserves their sequence. The agreement between the simulations results shown in figure S18 and the analytical distributions with limited number of binding sites (equation (3.6)) provides evidence that the mismatch between the analytical probability distribution and the simulation results in the main text section 3.3 is due to including sequence preservation on motor rebinding in those simulations.

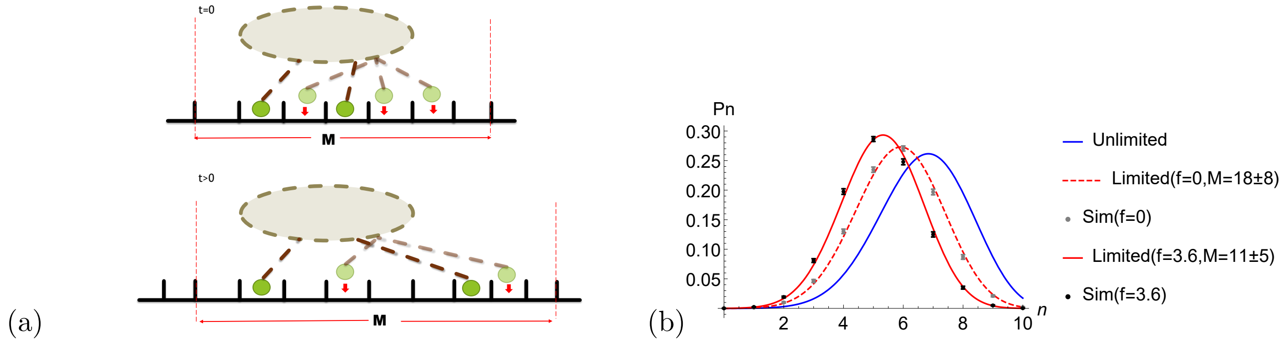

Figure S18: (a) Cartoon showing scenario B variable number of binding sites but without sequence preservation i.e. motors can bind to any available site such that they may swap positions by unbinding and re-binding. (b) Probability distribution of $N = 10$ non-processive motors at $f = 0$ and $f = f_s = 3.6$ attached on the same cargo. Simulation results (grey and black points), analytical equation (3.4) (dark blue line) and equation (3.6) using the average M number of binding sites found in the simulations (dashed and solid red lines). The parameter values used are $k_{\text{on}} = 20\,\text{s}^{-1}$, $k_{\text{off}} = 10\,\text{s}^{-1}$, $p = 22\,\text{s}^{-1}$, $q = 2.2\,\text{s}^{-1}$ and $\delta = 0.5$.

D. STALL FORCE FOR LARGE NUMBERS OF MOTORS

In figure S19 we show an extended version of figure 12 of the stall force against number of motors N up to $N = 100$. This additional data shows that the numerical result for non-processive motors with unlimited number of binding sites increases linearly for large number of motors ($N > 10$) with the same gradient as that for processive motors. This is also the case for binding sites limited to the average found in simulations.

The black circles in figure S19 show that for small number of motors the stall forces for non-processive Ncds extracted from our simulations correspond well with those those numerically calculated from the analytical solutions with the number of binding sites unlimited (green crosses) and limited (light blue crosses). However for $N > 3$ the results from our simulations are are lower than those obtained from the analytical expressions and remain lower for $N > 10$.

E. SINGLE MOTOR APPROXIMATION

For $q \ll pe^{-f/2}$, we can approximate equation (2.2) by $V_N \approx pe^{-f/2} - qe^{f/2}(q/p)^{N-1}$ which is the single motor case with the backwards stepping rate modified by the factor $(q/p)^{N-1}$. This can

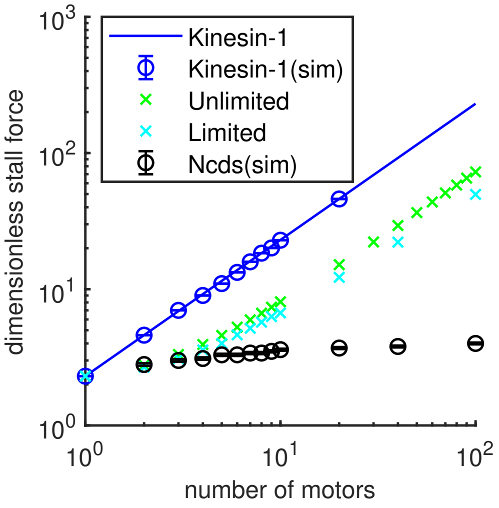

Figure S19: Extended version of main text figure 12 of dimensionless stall force against number of motors, N, including data for $10 \leq N \leq 100$ on a log-log plot. For processive kinesin-1 motors we calculate this analytically by setting equation (2.2) to zero (solid blue line). For non-processive Ncd motors we set equation (3.1) to zero and solve numerically using the distribution of bound motors with limited (equation (3.6), light blue crosses) and unlimited (equation (3.4) green crosses) binding sites. Simulation results are shown with circle symbols for parameter values $p = 100\,\mathrm{s}^{-1}$ and $q = 10\,\mathrm{s}^{-1}$ for kinesin-1 processive motors (blue circles) and $p = 22\,\mathrm{s}^{-1}$ $q = 2.2\,\mathrm{s}^{-1}$, $k_{\mathrm{on}} = 20\,\mathrm{s}^{-1}$ and $k_{\mathrm{off}} = 10\,\mathrm{s}^{-1}$ for Ncd non-processive motors (black circles).

reproduce an approximation to the curves seen in figure 3 (see figure S20) but has the disadvantage of only being valid for $q \ll pe^{-f/2}$ which is not well satisfied for our parameter values.

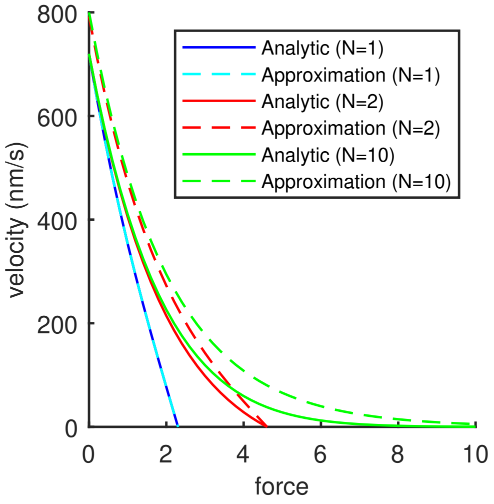

Figure S20: Force-velocity curves of $N = 1$ (blue), 2 (red) and 10 (green) processive motors using the full analytical expression, equation (2.2) (solid lines) and an approximate analytical expression $V_N \approx pe^{-f/2} - qe^{f/2}(q/p)^{N-1}$ which corresponds to a single motor with backwards stepping rate modified by the factor $(q/p)^{N-1}$ (dashed lines). The equivalent simulation points are shown on the main text figure 3.
